# Crystal structure of bis­(4-acetyl­pyridine-κ*N*)bis­(ethanol-κ*O*)bis­(thio­cyanato-κ*N*)manganese(II)

**DOI:** 10.1107/S2056989015004533

**Published:** 2015-03-11

**Authors:** Julia Werner, Inke Jess, Christian Näther

**Affiliations:** a Institut für Anorganische Chemie, Christian-Albrechts-Universität Kiel, Max-Eyth-Strasse 2, 24118 Kiel, Germany

**Keywords:** crystal structure, manganese(II), octa­hedral coordination, hydrogen bonding

## Abstract

In the crystal structure of the title compound, [Mn(NCS)_2_(C_7_H_7_NO)_2_(C_2_H_5_OH)_2_], the Mn^II^ atom is coordin­ated by two *N*-bonded thio­cyanate anions, two 4-acetyl­pyridine ligands, and two ethanol mol­ecules within a slightly distorted octa­hedron. The asymmetric unit consits of one manganese cation, located on a centre of inversion, one thio­cyanate anion, one 4-acetyl­pyridine ligand and one ethanol mol­ecule in general positions. The discrete complexes are connected by inter­molecular O—H⋯O hydrogen bonds between the alcohol OH group and the carbonyl O atom into chains parallel to [011].

## Related literature   

For a similar structure with thio­cyanato ligands in terminal coordination to a manganese(II) atom, see: Li *et al.* (2007 ▸).
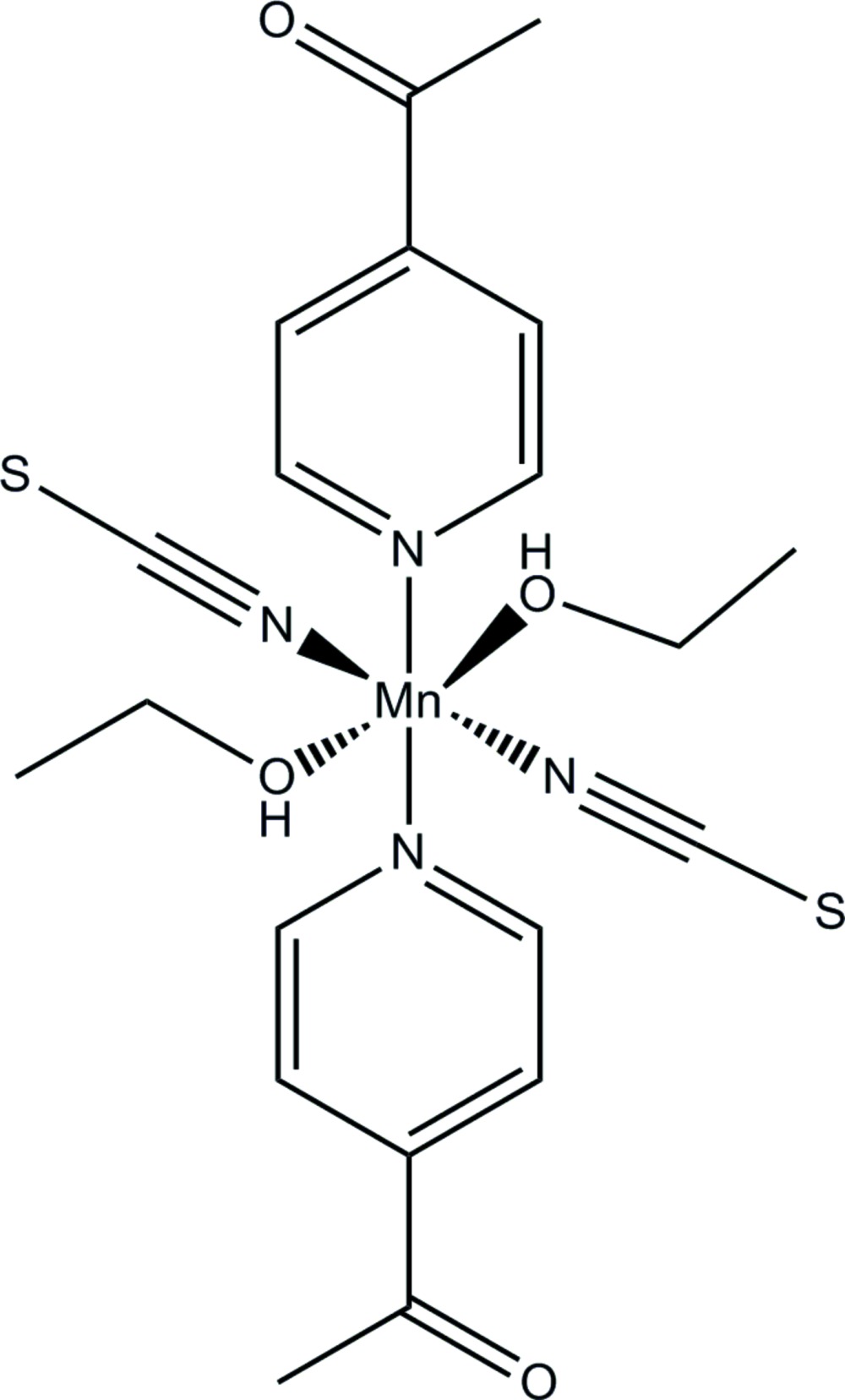


## Experimental   

### Crystal data  

[Mn(NCS)_2_(C_7_H_7_NO)_2_(C_2_H_6_O)_2_]
*M* *_r_* = 505.51
Triclinic, 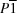

*a* = 6.9547 (7) Å
*b* = 9.7733 (9) Å
*c* = 10.1859 (9) Å
α = 117.449 (10)°
β = 94.978 (11)°
γ = 93.379 (11)°
*V* = 608.23 (11) Å^3^
*Z* = 1
Mo *K*α radiation
μ = 0.75 mm^−1^
*T* = 200 K
0.04 × 0.03 × 0.02 mm

### Data collection  

Stoe IPDS-1 diffractometer
Absorption correction: numerical (*X-SHAPE* and *X-RED32*; Stoe & Cie, 2008 ▸) *T*_min_ = 0.966, *T*_max_ = 0.977
6535 measured reflections
2583 independent reflections
2163 reflections with *I* > 2σ(*I*)
*R*_int_ = 0.039

### Refinement  

*R*[*F* ^2^ > 2σ(*F* ^2^)] = 0.033
*wR*(*F* ^2^) = 0.088
*S* = 1.04
2583 reflections
144 parameters
H-atom parameters constrained
Δρ_max_ = 0.26 e Å^−3^
Δρ_min_ = −0.35 e Å^−3^

### 

Data collection: *X-AREA* (Stoe & Cie, 2008 ▸); cell refinement: *X-AREA*; data reduction: *X-AREA*; program(s) used to solve structure: *SHELXS97* (Sheldrick, 2008 ▸); program(s) used to refine structure: *SHELXL2013* (Sheldrick, 2015 ▸); molecular graphics: *XP* in *SHELXTL* (Sheldrick, 2008 ▸) and *DIAMOND* (Brandenburg, 1999 ▸); software used to prepare material for publication: *publCIF* (Westrip, 2010 ▸).

## Supplementary Material

Crystal structure: contains datablock(s) I, global. DOI: 10.1107/S2056989015004533/wm5131sup1.cif

Structure factors: contains datablock(s) I. DOI: 10.1107/S2056989015004533/wm5131Isup2.hkl

**Fig. 1.**
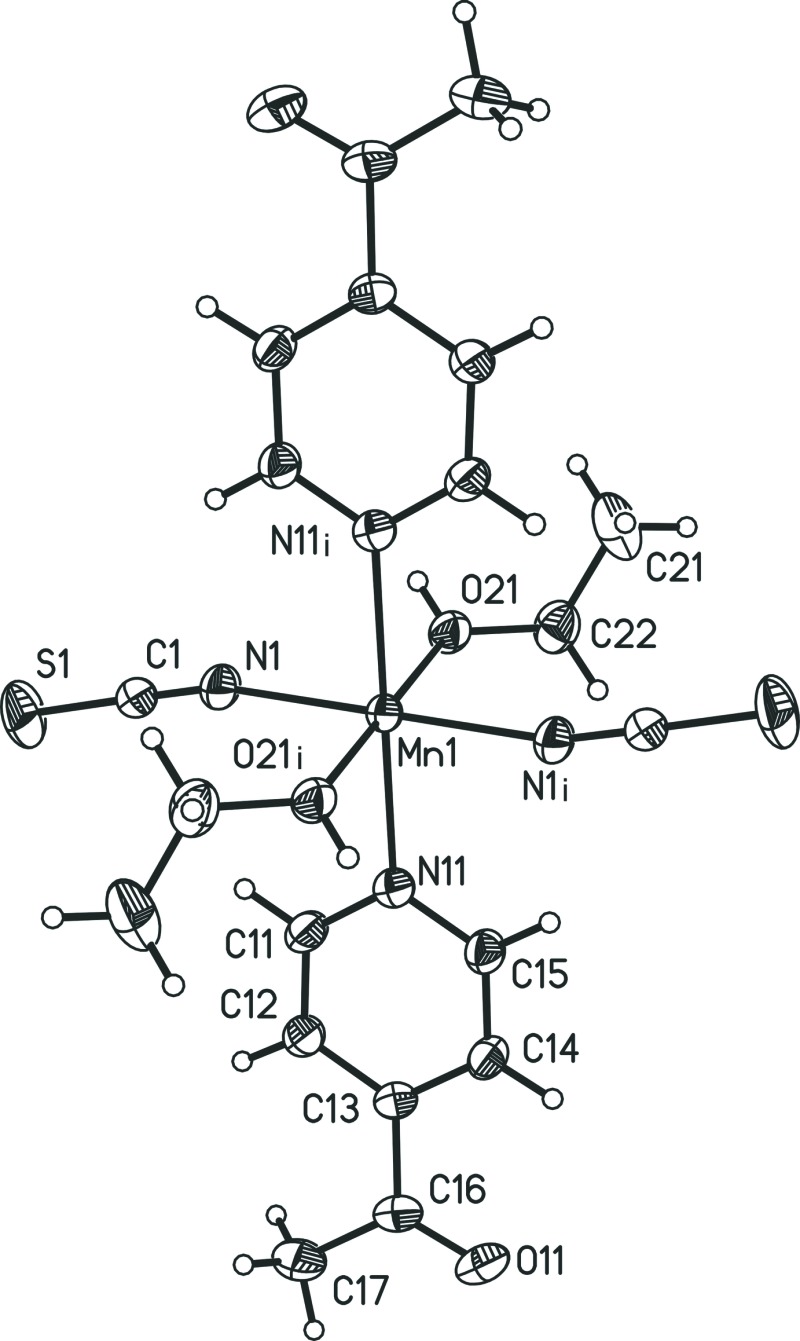
The coordination environment of the MnII atom in the title compound. Displacement ellipsoids are drawn at the 50% probability level. [Symmetry code: (i) -x + 2, -y + 1, -z + 1.]

II x y z . DOI: 10.1107/S2056989015004533/wm5131fig1.tif
The coordination environment of the Mn^II^ atom in the title compound. Displacement ellipsoids are drawn at the 50% probability level. [Symmetry code: (i) −*x* + 2, −*y* + 1, −*z* + 1.]

**Fig. 2.**
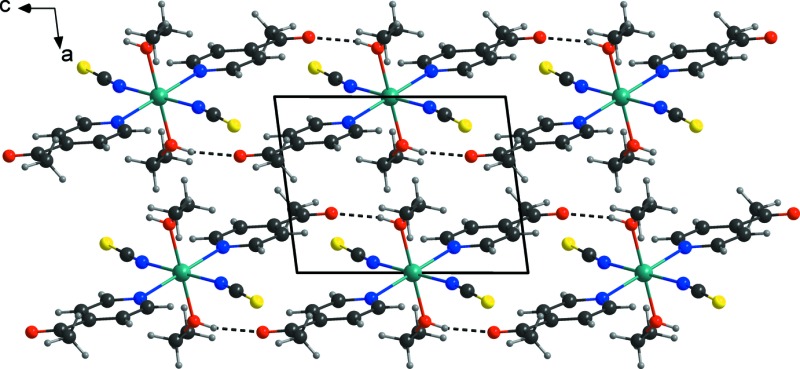
Crystal structure of the title compound in a view along [010]. Hydrogen bonds are indicated by dashed lines.

. DOI: 10.1107/S2056989015004533/wm5131fig2.tif
Crystal structure of the title compound in a view along [010]. Hydrogen bonds are indicated by dashed lines.

CCDC reference: 1052202

Additional supporting information:  crystallographic information; 3D view; checkCIF report

## Figures and Tables

**Table 1:** Hydrogen-bond geometry (, )

*D*H*A*	*D*H	H*A*	*D* *A*	*D*H*A*
O21H1*O*1O11^i^	0.84	1.95	2.7714(17)	164

Symmetry code: (i) 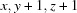.

## supplementary crystallographic information

### S1. Synthesis and crystallization

MnSO_4_·H_2_O was purchased from Merck; 4-acetyl­pyridine and Ba(NCS)_2_·3H_2_O were purchased from Alfa Aesar. Mn(NCS)_2_ was synthesized by stirring 17.97 g (58.44 mmol) Ba(NCS)_2_·3H_2_O and 9.88 g (58.44 mmol) MnSO_4_·H_2_O in 400 ml water at room temperature for three hours. The white residue of BaSO_4_ was filtered off and the solvent evaporated using a rotary evaporator. The homogeneity of the product was investigated by X-ray powder diffraction and elemental analysis. The title compound was prepared by the reaction of 42.8 mg (0.25 mmol) Mn(NCS)_2_ and 55.1 µl (0.50 mmol) 4-acetyl­pyridine in 1.5 ml ethanol at room temperature. After several days, suitable crystals of the title compound were obtained.

### S2. Refinement

The C-bound H atoms were positioned with idealized geometry (methyl H atoms allowed to rotate but not to tip) and were refined with *U*_iso_(H) = 1.2*U*_eq_(C) (1.5 for methyl H atoms) using a riding model with C—H = 0.95 Å for aromatic, C—H = 0.99 Å for methyl­ene and C—H = 0.98 Å for methyl H atoms. The O-bound H atom was located in a difference map. Its bond length was set to a value of 0.84 Å and it was refined with *U*_iso_(H) = 1.5*U*_eq_(O) using a riding model.

### Crystal data

**Table:**

[Mn(NCS)_2_(C_7_H_7_NO)_2_(C_2_H_6_O)_2_]	*Z* = 1
*M**_r_* = 505.51	*F*(000) = 263
Triclinic, *P*1	*D*_x_ = 1.380 Mg m^−^^3^
*a* = 6.9547 (7) Å	Mo *K*α radiation, λ = 0.71073 Å
*b* = 9.7733 (9) Å	Cell parameters from 6535 reflections
*c* = 10.1859 (9) Å	θ = 2.4–27.0°
α = 117.449 (10)°	µ = 0.75 mm^−^^1^
β = 94.978 (11)°	*T* = 200 K
γ = 93.379 (11)°	Block, colorless
*V* = 608.23 (11) Å^3^	0.04 × 0.03 × 0.02 mm

### Data collection

**Table:**

Stoe IPDS-1 diffractometer	2163 reflections with *I* > 2σ(*I*)
phi scans	*R*_int_ = 0.039
Absorption correction: numerical (*X-SHAPE* and *X-RED32*; Stoe & Cie, 2008)	θ_max_ = 27.0°, θ_min_ = 2.4°
*T*_min_ = 0.966, *T*_max_ = 0.977	*h* = −8→8
6535 measured reflections	*k* = −12→12
2583 independent reflections	*l* = −13→13

### Refinement

**Table:**

Refinement on *F*^2^	Hydrogen site location: mixed
Least-squares matrix: full	H-atom parameters constrained
*R*[*F*^2^ > 2σ(*F*^2^)] = 0.033	*w* = 1/[σ^2^(*F*_o_^2^) + (0.0565*P*)^2^] where *P* = (*F*_o_^2^ + 2*F*_c_^2^)/3
*wR*(*F*^2^) = 0.088	(Δ/σ)_max_ < 0.001
*S* = 1.04	Δρ_max_ = 0.26 e Å^−^^3^
2583 reflections	Δρ_min_ = −0.35 e Å^−^^3^
144 parameters	Extinction correction: *SHELXL2013* (Sheldrick, 2015), Fc^*^=kFc[1+0.001xFc^2^λ^3^/sin(2θ)]^-1/4^
0 restraints	Extinction coefficient: 0.069 (8)

### Special details

**Table:**

Geometry. All esds (except the esd in the dihedral angle between two l.s. planes) are estimated using the full covariance matrix. The cell esds are taken into account individually in the estimation of esds in distances, angles and torsion angles; correlations between esds in cell parameters are only used when they are defined by crystal symmetry. An approximate (isotropic) treatment of cell esds is used for estimating esds involving l.s. planes.

### Fractional atomic coordinates and isotropic or equivalent isotropic displacement parameters (Å^2^)

**Table:**

	*x*	*y*	*z*	*U*_iso_*/*U*_eq_	
Mn1	1.0000	0.5000	0.5000	0.02081 (14)	
N1	0.9402 (2)	0.42929 (18)	0.66517 (15)	0.0300 (3)	
C1	0.8976 (2)	0.35987 (19)	0.72660 (17)	0.0245 (3)	
S1	0.83508 (10)	0.25963 (7)	0.80913 (7)	0.05345 (19)	
N11	0.8542 (2)	0.25989 (15)	0.30663 (15)	0.0250 (3)	
C11	0.8030 (3)	0.1414 (2)	0.33223 (19)	0.0339 (4)	
H11	0.8081	0.1609	0.4329	0.041*	
C12	0.7431 (3)	−0.0080 (2)	0.22080 (19)	0.0338 (4)	
H12	0.7099	−0.0886	0.2451	0.041*	
C13	0.7325 (2)	−0.03842 (18)	0.07316 (17)	0.0241 (3)	
C14	0.7813 (3)	0.0837 (2)	0.04487 (18)	0.0276 (4)	
H14	0.7738	0.0676	−0.0549	0.033*	
C15	0.8413 (3)	0.2296 (2)	0.16300 (18)	0.0278 (4)	
H15	0.8749	0.3123	0.1417	0.033*	
C16	0.6741 (3)	−0.19848 (19)	−0.05499 (18)	0.0285 (4)	
C17	0.6365 (3)	−0.3314 (2)	−0.0243 (2)	0.0417 (5)	
H17A	0.6077	−0.4275	−0.1188	0.063*	
H17B	0.5254	−0.3158	0.0322	0.063*	
H17C	0.7515	−0.3384	0.0341	0.063*	
O11	0.6621 (2)	−0.21465 (16)	−0.18155 (14)	0.0409 (3)	
C21	0.6624 (4)	0.8271 (3)	0.4935 (3)	0.0549 (6)	
H21A	0.5856	0.8597	0.4296	0.082*	
H21B	0.6313	0.8831	0.5958	0.082*	
H21C	0.8010	0.8504	0.4921	0.082*	
C22	0.6154 (3)	0.6558 (2)	0.4366 (2)	0.0367 (4)	
H22A	0.4748	0.6326	0.4363	0.044*	
H22B	0.6445	0.5999	0.3325	0.044*	
O21	0.72349 (18)	0.60072 (14)	0.52571 (12)	0.0291 (3)	
H1O1	0.6854	0.6446	0.6101	0.044*	

### Atomic displacement parameters (Å^2^)

**Table:**

	*U*^11^	*U*^22^	*U*^33^	*U*^12^	*U*^13^	*U*^23^
Mn1	0.0266 (2)	0.01896 (19)	0.01844 (18)	0.00180 (12)	0.00503 (13)	0.00984 (14)
N1	0.0409 (9)	0.0290 (7)	0.0236 (7)	0.0005 (6)	0.0079 (6)	0.0150 (6)
C1	0.0280 (8)	0.0232 (7)	0.0217 (7)	0.0021 (6)	0.0039 (6)	0.0099 (6)
S1	0.0678 (4)	0.0535 (4)	0.0652 (4)	0.0037 (3)	0.0157 (3)	0.0489 (3)
N11	0.0295 (7)	0.0219 (7)	0.0220 (6)	0.0014 (5)	0.0027 (5)	0.0091 (6)
C11	0.0525 (12)	0.0266 (8)	0.0206 (7)	−0.0014 (8)	0.0051 (7)	0.0099 (7)
C12	0.0539 (12)	0.0225 (8)	0.0245 (8)	−0.0016 (8)	0.0056 (8)	0.0111 (7)
C13	0.0237 (8)	0.0241 (7)	0.0219 (7)	0.0041 (6)	0.0051 (6)	0.0080 (6)
C14	0.0323 (9)	0.0305 (8)	0.0194 (7)	0.0012 (7)	0.0039 (6)	0.0114 (7)
C15	0.0338 (9)	0.0267 (8)	0.0240 (7)	−0.0015 (7)	0.0026 (7)	0.0136 (7)
C16	0.0273 (8)	0.0258 (8)	0.0254 (8)	0.0060 (7)	0.0056 (7)	0.0053 (7)
C17	0.0550 (13)	0.0229 (9)	0.0374 (10)	−0.0008 (8)	0.0026 (9)	0.0069 (8)
O11	0.0527 (8)	0.0372 (7)	0.0221 (6)	0.0057 (6)	0.0073 (6)	0.0043 (5)
C21	0.0604 (15)	0.0522 (13)	0.0762 (16)	0.0160 (11)	0.0178 (13)	0.0477 (13)
C22	0.0325 (9)	0.0459 (11)	0.0341 (9)	0.0083 (8)	0.0003 (7)	0.0210 (9)
O21	0.0336 (6)	0.0315 (6)	0.0238 (5)	0.0115 (5)	0.0080 (5)	0.0128 (5)

### Geometric parameters (Å, º)

**Table:**

Mn1—N1^i^	2.1543 (15)	C14—C15	1.385 (2)
Mn1—N1	2.1543 (15)	C14—H14	0.9500
Mn1—O21^i^	2.1928 (12)	C15—H15	0.9500
Mn1—O21	2.1929 (12)	C16—O11	1.219 (2)
Mn1—N11^i^	2.3508 (14)	C16—C17	1.484 (3)
Mn1—N11	2.3508 (14)	C17—H17A	0.9800
N1—C1	1.157 (2)	C17—H17B	0.9800
C1—S1	1.6211 (18)	C17—H17C	0.9800
N11—C11	1.334 (2)	C21—C22	1.502 (3)
N11—C15	1.346 (2)	C21—H21A	0.9800
C11—C12	1.383 (2)	C21—H21B	0.9800
C11—H11	0.9500	C21—H21C	0.9800
C12—C13	1.386 (2)	C22—O21	1.435 (2)
C12—H12	0.9500	C22—H22A	0.9900
C13—C14	1.381 (3)	C22—H22B	0.9900
C13—C16	1.505 (2)	O21—H1O1	0.8400
			
N1^i^—Mn1—N1	180.0	C13—C14—H14	120.2
N1^i^—Mn1—O21^i^	88.59 (5)	C15—C14—H14	120.2
N1—Mn1—O21^i^	91.41 (5)	N11—C15—C14	123.05 (16)
N1^i^—Mn1—O21	91.41 (5)	N11—C15—H15	118.5
N1—Mn1—O21	88.59 (5)	C14—C15—H15	118.5
O21^i^—Mn1—O21	180.0	O11—C16—C17	122.10 (16)
N1^i^—Mn1—N11^i^	91.13 (5)	O11—C16—C13	118.40 (17)
N1—Mn1—N11^i^	88.87 (5)	C17—C16—C13	119.49 (16)
O21^i^—Mn1—N11^i^	92.35 (5)	C16—C17—H17A	109.5
O21—Mn1—N11^i^	87.65 (5)	C16—C17—H17B	109.5
N1^i^—Mn1—N11	88.87 (5)	H17A—C17—H17B	109.5
N1—Mn1—N11	91.13 (5)	C16—C17—H17C	109.5
O21^i^—Mn1—N11	87.65 (5)	H17A—C17—H17C	109.5
O21—Mn1—N11	92.35 (5)	H17B—C17—H17C	109.5
N11^i^—Mn1—N11	180.0	C22—C21—H21A	109.5
C1—N1—Mn1	164.93 (13)	C22—C21—H21B	109.5
N1—C1—S1	178.67 (16)	H21A—C21—H21B	109.5
C11—N11—C15	116.73 (14)	C22—C21—H21C	109.5
C11—N11—Mn1	121.49 (11)	H21A—C21—H21C	109.5
C15—N11—Mn1	121.25 (11)	H21B—C21—H21C	109.5
N11—C11—C12	123.87 (16)	O21—C22—C21	112.19 (18)
N11—C11—H11	118.1	O21—C22—H22A	109.2
C12—C11—H11	118.1	C21—C22—H22A	109.2
C11—C12—C13	118.96 (17)	O21—C22—H22B	109.2
C11—C12—H12	120.5	C21—C22—H22B	109.2
C13—C12—H12	120.5	H22A—C22—H22B	107.9
C14—C13—C12	117.87 (15)	C22—O21—Mn1	130.79 (11)
C14—C13—C16	119.69 (15)	C22—O21—H1O1	105.5
C12—C13—C16	122.44 (16)	Mn1—O21—H1O1	120.2
C13—C14—C15	119.50 (15)		

Symmetry code: (i) −*x*+2, −*y*+1, −*z*+1.

### Hydrogen-bond geometry (Å, º)

**Table:**

*D*—H···*A*	*D*—H	H···*A*	*D*···*A*	*D*—H···*A*
O21—H1*O*1···O11^ii^	0.84	1.95	2.7714 (17)	164

Symmetry code: (ii) *x*, *y*+1, *z*+1.
