# Supplementary figures and images for: Crystal structure of bis­(4-acetyl­pyridine-κN)bis­(ethanol-κO)bis­(thio­cyanato-κN)manganese(II)
Source: Acta Crystallogr E Crystallogr Commun. 2015 Mar 11;71(Pt 4):m81–2. doi: 10.1107/S2056989015004533 (PMC4438791; doi:10.1107/S2056989015004533)

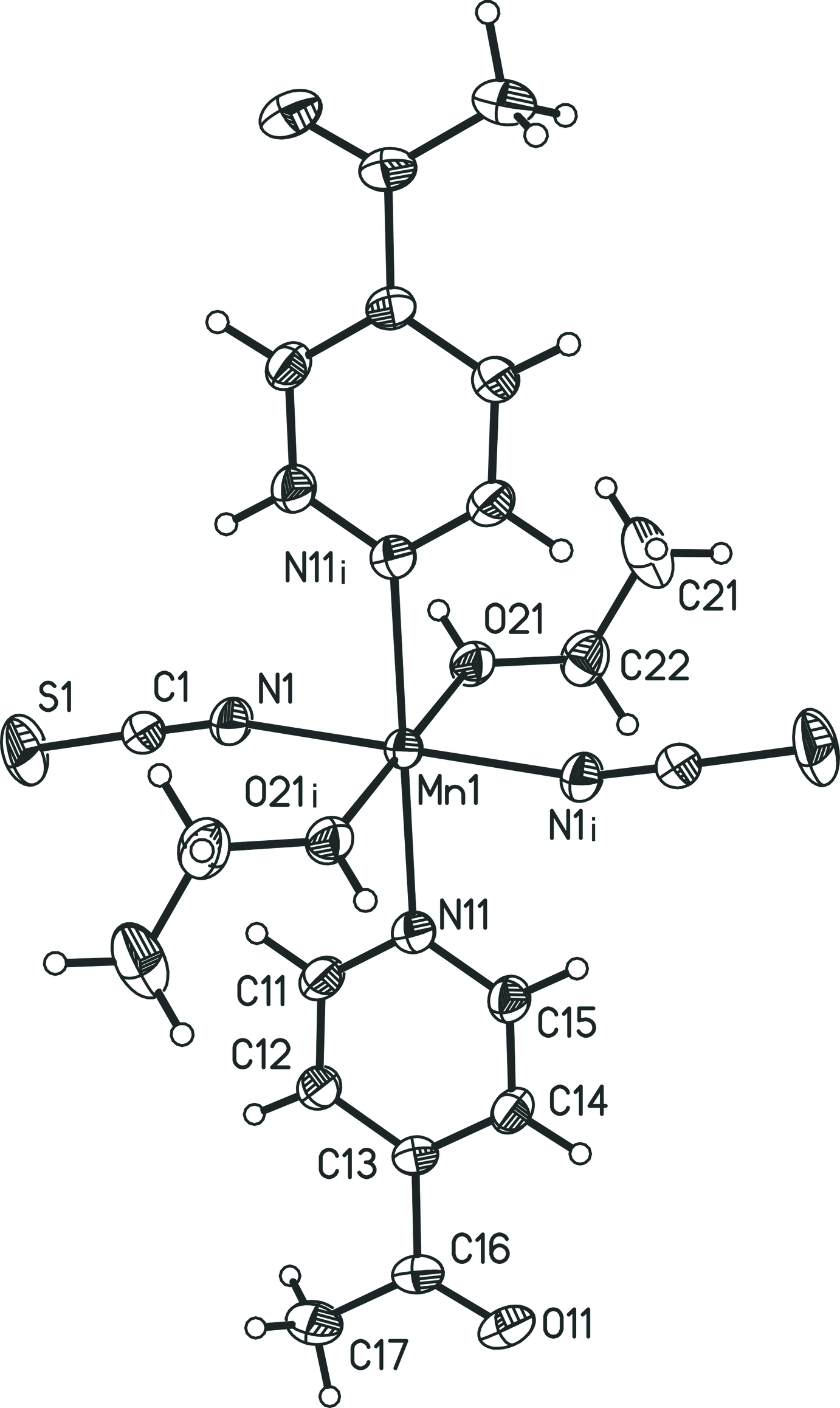

Supplement: Supplementary file 3 [file e-71-00m81-fig1.tif]

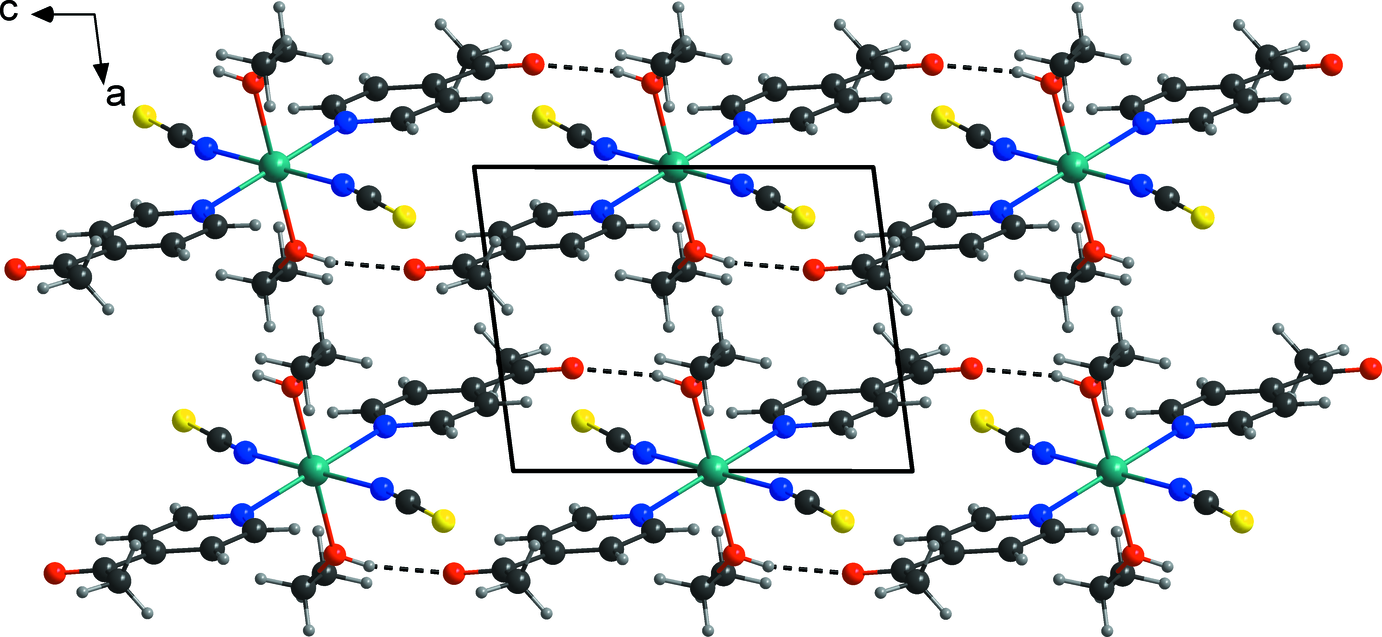

Supplement: Supplementary file 4 [file e-71-00m81-fig2.tif]
